# Effect of multicomponent exercise in cognitive impairment: a systematic review and meta-analysis

**DOI:** 10.1186/s12877-022-03302-1

**Published:** 2022-07-25

**Authors:** Luis Carlos Venegas-Sanabria, Iván Cavero-Redondo, Vicente Martínez-Vizcaino, Carlos Alberto Cano-Gutierrez, Celia Álvarez-Bueno

**Affiliations:** 1 Universidad de Castilla-La Mancha, Health and Social Research Center, Cuenca, Spain; 2 Instituto Rosarista para el Estudio del Envejecimiento y la Longevidad, Escuela de Medicina y Ciencias de la Salud, Universidad del Rosario, Bogotá, Colombia; 3 Hospital Universitario Mayor, Méderi, Bogotá, Colombia; 4 Rehabilitation in Health Research Center (CIRES), Universidad de Las Américas, Santiago, Chile; 5 Facultad de Enfermería de Cuenca, C/ Santa Teresa Jornet, s/n. Cuenca, Spain; 6 Facultad de Ciencias de la Salud, Universidad Autónoma de Chile, Santiago, Chile; 7 Aging Institute, Medicine School, Pontificia Universidad Javeriana, Bogotá, Colombia; 8 Department of Internal Medicine, Geriatric Unit, Hospital Universitario San Ignacio, Bogotá, Colombia; 9 Universidad Politécnica Y Artística del Paraguay, Asunción, Paraguay

**Keywords:** Cognitive impairment, Physical activity, Non-pharmacological treatment, Meta-analysis

## Abstract

**Background:**

Multicomponent physical exercise is the most recommended type of physical intervention in older adults. Experimental data suggest the relevance of the muscle-brain axis and the relationship between muscle contraction and release of brain-derived neurotrophic factor, however, the impact of this relationship on cognition remains unclear, especially in people with diagnosis of cognitive impairment. This study assesses the effect of multicomponent physical exercise on global cognition in people with mild cognitive impairment or dementia.

**Methods:**

Randomized controlled trials published until January 2021 were searched across three electronic databases (PubMed, Scopus, and Cochrane Database). Data about exercises included in the multicomponent intervention (endurance, strength, balance, or flexibility), the inclusion of aerobic exercise, and the change in global cognition were extracted. The effect size was represented as a standardized mean difference. Risk of bias was assessed by the RoB2 tool.

**Results:**

A total of 8 studies were included. The overall effect size suggested an effect of multicomponent exercise on global cognition. However, the subgroup analysis showed an effect only when aerobic exercise was included in the intervention. No effect when mild cognitive impairment and dementia were assessed separately was found.

**Conclusion:**

This study suggests that multicomponent physical exercise could have an effect on global cognition in people with mild cognitive impairment or dementia only when aerobic exercise is included in the intervention. Our results support the inclusion of structured physical exercise programs in the management of people with cognitive impairment.

**Supplementary Information:**

The online version contains supplementary material available at 10.1186/s12877-022-03302-1.

## Background

In recent decades we are living a paradox, we have experienced a steady increase in life expectancy, which has been accompanied by an increase in chronic conditions and associated with a high functional cost [1]. This functional cost not only brings health consequences, but also a huge socioeconomic impact. Few conditions exemplify this picture as cognitive impairment, a condition that affect almost fifty million people worldwide according to the latest World Health Organization (WHO) report [2]. This report highlights the importance of implementing an international plan for risk factors education, assessment and management early diagnosis, targeted treatment, caregiver assistance, and dementia-related research. Cognitive decline has been associated with an increase in the risk of functional decline, falls, need of long-term-care, care given burden, and an increase in the direct and indirect cost related with the attention, among others [3]. However, although most developed countries have designed and implemented national strategies to reduce the burden of dementia, their impact has been slightly modified [4, 5], which highlights the relevance of the implementation of any intervention that could reduce this burden.

Currently, there is not enough evidence available for disease-modifying pharmacological treatments [6], and the benefits of these treatments on cognition are limited [7, 8]. For this reason, non-pharmacological treatments to manage dementia and mild cognitive impairment (MCI) are currently a relevant research topic [9]. Research on physical activity and physical exercise, as part of non-pharmacological interventions, have been of interest not only for their effects on physical performance but also for their effects on cognitive function. Evidence provided by systematic reviews and meta-analyses suggests that physical exercise could improve global cognition in people with MCI or dementia [10–12]. A network meta-analysis that compared the effect of non-pharmacological interventions on cognition in people with Alzheimer´s disease or MCI, showed that physical exercise could produce a significant improvement compared with other interventions [13].

Among the effects of the different types of physical exercise on the cognition of people with cognitive impairment, some questions remain unanswered, such as the effect of multicomponent physical exercises. This type of exercise includes endurance, strength, balance, and flexibility training [14], and is the most recommended exercise for older people showing positive effects on functional decline during hospitalization [15], frailty [16], and sarcopenia [17]. From this perspective, the role of multicomponent physical exercise over cognition has aroused interest. Increase evidence suggests the existence of a muscle-brain axis that could explain the exercise-induced neuroprotection, which may be related to the improvement in mitochondrial function by exercise in skeletal muscle [18]. In addition, muscle contraction has been related to an increase in the expression of the brain-derived neurotrophic factor (BDNF) [19]. In general, physical exercise increases BDNF in neurodegenerative diseases regardless of the duration, or intensity of the intervention [20]. However, when the effect of strength exercise is assessed, no association was found [21].

The effects of multicomponent physical exercise on cognitive function, especially in people diagnosed with cognitive impairment, are still unclear. Therefore, the aim of this systematic review and meta-analysis is to estimate the effect of multicomponent physical exercise on global cognition in people with MCI or dementia.

## Methods

### Protocol and registration

The protocol of this systematic review was registered at the International Prospective Register of Systematic Reviews (PROSPERO), with the register identification CRD42020184660. In addition, the protocol was approved by the Ethics Committee of the Pontificia Universidad Javeriana and the Hospital Universitario San Ignacio.

### Search strategy

A search was performed on MEDLINE (via PubMed), Scopus, and Cochrane Database up to January 2021. The search was aimed to identify published randomized controlled trials assessing the effect of multicomponent physical exercise on global cognition in people with dementia and MCI. Global cognition was considered the outcome because it is the best measurement of the cognitive function in patients with dementia and MCI and is the most common way to assess cognition in the included randomized controlled trials. We examined the reference list of eligible studies to expand the search. The complete MEDLINE search strategy is displayed in Supplementary material (Supplementary Table S1). The search was performed to identify any type of physical exercise that could be part of the multicomponent intervention according with the definition proposed by Cress et al [14]. Additionally, different types of cognitive impairment were included in the search strategy to try of encompass most of the etiologies.

### Inclusion criteria

The studies included in this systematic review met the following inclusion criteria: (i) Participants: adult participants with dementia or MCI secondary to either. (ii) Intervention: multicomponent physical exercise defined as an exercise program including endurance, strength, balance, or flexibility [14]. We included studies developing any intervention program that included at least two of the above-mentioned exercises without other intervention (e.g., cognitive interventions), and regardless of the inclusion of aerobic exercise as part of the intervention. (iii) Outcome: global cognition measured by any validate neuropsychological test. (iv) Control: any control was accepted except those with a physical activity component. If the control intervention included a component of cognitive training or stimulation, both total and partially, the RCT was included in the analysis. (v) Type of study: randomized controlled trials. We only included in this review studies in English and Spanish. No exclusion criteria were considered.

### Study identification and data extraction

After defining the search strategy, all records were imported into the reference management system (Mendeley, desktop version 1.19.4) to exclude duplicate records. Two authors (LVS and CAB) independently performed the search literature and data extraction, with the intervention of a third author in case of disagreement (ICR). The following data were extracted from the selected studies using an ad-hoc form: author, publication year, country, sample size, losses, female proportion, mean age in control and intervention group, type of cognitive impairment, types of physical exercises included in the intervention, length of the intervention (weeks), number of sessions per week, duration per session (minutes), type of control group, the intensity of the multicomponent exercise (as reported by each study), cognitive assessment tool and effect on global cognition.

### Risk of bias

The risk of bias was assessed independently by two authors using the revised Cochrane risk-of-bias tool for randomized trials (RoB2) [22]. The RoB2 included assessment of six domains: randomization process, derivation for intended interventions, missing outcome data, measurement of the outcome, selection of the reported results, and overall results. Each domain was rated as low, moderate, and high risk of bias. To report on the risk of bias, a graphical representation was used.

### Data analysis

The effect size (ES) and 95% confidence intervals (CIs) for the effect of physical exercise programs on global cognition were calculated using the Cohen’s d index. A pooled ES was estimated using a random-effects model based on the Der Simonian and Laird method [23]. We consider the ES classified as proposed by Cohen et al. [24]; small effect (0.2), moderate effect (0.5), and large effect (0.8). Inconsistence across studies was assessed using the I^2^ statistic [25], whose values were considered as follows: not important (0%–40%), moderate (30–60%), substantial (50–90%), and considerable (75–100%). Moreover, the corresponding p value were also considered. To determine the size and clinical relevance of heterogeneity, τ^2^ statistic was calculated and interpreted as low when τ^2^ was lower than 0.04, moderate when was from 0.04 to 0.14 and as substantial when it was from 0.14 to 0.40 [26]. In case of high heterogeneity, two subgroup analyses were developed: i) by type of cognitive impairment, distinguishing between patients with MCI and dementia, and ii) by the inclusion of aerobic exercise in the intervention. When global cognition was measured using more than one scale, a pooled ES was calculated. To define the effect of individual studies in the overall effect size a sensitive analysis was performed.

The small study effect was graphically assessed using a funnel plot, additionally, the Egger test was estimated. Finally, the trim-and-fill analysis was used to estimate the number of studies needed to remove publication bias, and to estimate the ES without publication bias. Stata/IC software, version 16.1 for Windows was used for statistical analysis.

## Results

### Study identification

**Fig. 1:**
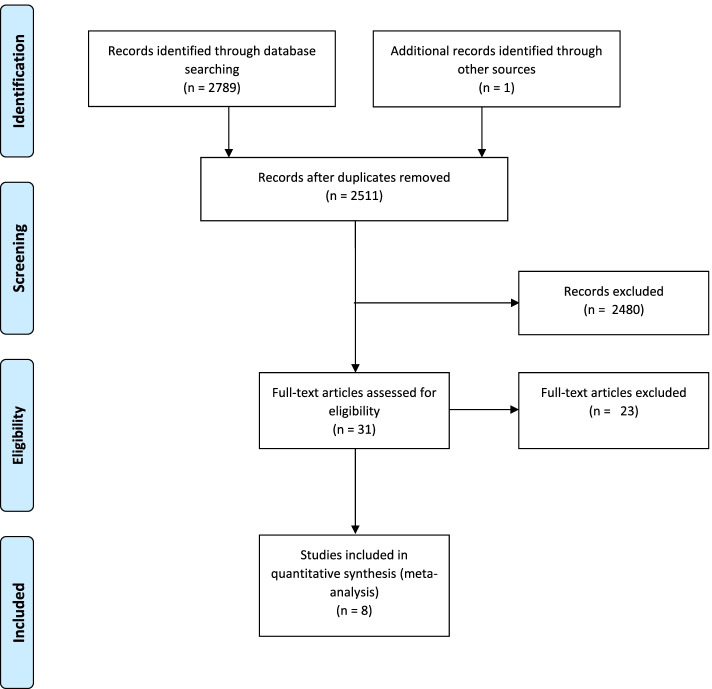
Flow diagram for searching and selection of the included studies

A total of 2789 records were found following the search strategy. After removing duplicates, and reviewing the title and abstract, 31 relevant papers were chosen for full reading. Finally, only 8 articles met the inclusion criteria (Fig. 1). The excluded articles and the reasons for exclusion are reported in the Supplementary Table S2.

### Characteristics of included studies

Eight studies assessing the effect of multicomponent physical exercise on global cognition in people with dementia and MCI [27–34] were included in the review. The studies included 367 participants from seven countries: France [29, 31], Spain [27], Brazil [28], Croatia [30], Korea [32], United States [33], and Japan [34]. The mean age was between 77.7 and 77.9 years in the intervention and control group, respectively, and women accounted for 70.3%. Five studies [28, 29, 31–33] included only participants with dementia and three with MCI [27, 30, 34].

Regarding multicomponent physical exercise interventions, all studies included at least two types of exercise considered in the definition of multicomponent physical exercise [14]. All studies included balance exercises, four included flexibility exercises [27, 31–33] and one did not include strength training [31]. Aerobic exercise was part of the intervention in six studies [27, 29, 31–34]. The intensity of physical activity was considered low to moderate in three papers [27, 31, 34], moderate in two [29, 33], and low in one [32], additionally, two studies did not report on exercise intensity [28, 30]. The length of the intervention programs was 8 to 52 weeks, including 1 to 7 sessions per week, and 30 to 90 min per session.

The Mini-Mental State Examination [27–29, 32, 34] was the most widely used cognitive measurement tool, other tools included were Montreal Cognitive Assessment (MoCA) [30], Brief Cognitive Screening Battery (BCSB) [28], Rapid Evaluation of Cognitive Function (ERFC) [31], Boston Naming Test (BNT) [33], Hopkins Verbal Learning Test (HVLT) [33], and Alzheimer’s Disease Assessment Scale-Cognitive (ADAS-Cog) [34]. Table 1 summarizes the studies included in the review.

**Table 1:** Characteristics of the included studies

Author, year and country	Sample size (Lost)	Age (mean ± SD)	Women (%)	Cognitive impairment type	Physical activity intensity	Program duration (weeks)	Sessions per week	Session duration (minutes)	Physical activity included	Control type	Results (SMD)
Bisbe et al., 2020 Spain	I: 18 (4) C: 18 (1)	I: 77.2(5.1) C: 72.8(5.6)	I: 50 C: 47.1	aMCI	Low to moderate	12	2	60	Aerobic Strength Balance Flexibility	Choreographed dances	MMSE: 0.23(-0.39, 0.84)
Christofoletti et al., 2008 Brazil	I: 17 (5) C: 20 (3)	I: 72.9(2.3) C: 79.4(2.0)	I: 70.5 C: 70	Dementia	NR	26	3	60	Strength Balance	Not motor intervention	MMSE: 0.05(-0.590, 0.703) BCSB test *Nomination:* 0.265(-0.383, 0.915) *Incidental memory:* 0.133(-0.513, 0.781) *Immediate memory:* -1.288(-1.288, -0.578) *Learning memory:* -0.926(-1.606, -0.246) *Delayed memory:* -0.133(-0.781, 0.513) *CDT:* 0.286(-0.363, 0.936) *Verbal fluency*: -0.872(-1.548, -0.195) *Recognition*: -0.090(-0.090, 0.556)
de Souto Barreto et al., 2017 France	I: 48 (4) C: 50 (3)	I: 88.3(5.1) C: 86.9(5.8)	I: 93.2 C: 76.6	Dementia	Moderate	24	2	60	Aerobic Strength Balance Coordination	Social intervention	MMSE: 0.038(-0.373, 0.450)
Greblo et al., 2017 Croatia	I: 14 (NR) C: 14 (NR)	70.4(3.9)	I: 100 C:100	MCI	NR	8	3	30	Strength Balance	Pilates	MoCA: 0.88(0.077, 1.687)
Kemoun et al., 2010 France	I: 20 (4) C: 18 (3)	I: 82(5.8) C: 81.7(5.1)	I: 75 C: 73.3	Alzheimer-type dementia	Low to moderate	15	1	60	Aerobic Balance Flexibility	Usual care	ERFC: 0.889(0.151, 1.628)
Kwak et al., 2008 Korea	I: 15 (NR) C: 15 (NR)	I: 79.6(6.6) C: 82.2(7.0)	I: 100 C: 100	Dementia	Low	52	1	40	Aerobic Strength Balance Flexibility	NR	MMSE: 1.02(0.267, 1.791)
Steinberg et al., 2009 US	I: 14 (NR) C:13 (NR)	I: 76.5(3.9) C: 74(8.1)	I: 71.1 C: 69.2	Alzheimer-type dementia	Moderate	12	7	NR	Aerobic Strength Balance Flexibility	Home safety assessment	BMT: 0.087(-0.668, 0.842) HVLT: 0.013(-0.741, 0.768)
Suzuki et al., 2013 Japan	I: 50 (3) C: 50 (5)	I: 74.8 (7.4) C: 75.8 (6.1)	I: 50 C: 48	MCI	Low to moderate	26	2	90	Aerobic Strength Balance	Education about health promotion	MMSE: 0.221(-0.189, 0.631) ADAS-Cog: -0.303(-0.714, 0.108)

*MMSE* Mini-mental state examination, *BCSB* Brief cognitive screening battery, *MoCA* Montreal *Cognitive Assessment*, *ERFC* Rapid evaluation of cognitive function, *BNT* Boston naming test, *HVLT* Hopkins verbal learning test, *Adas-Cog* Alzheimer’s disease assessment scale-cognitive, *NR* Non reported, *MCI* Mild cognitive impairment, *aMCI* Amnestic mild cognitive impairment

### Risk of bias

The risk of bias was assessed using the revised Cochrane risk-of-bias tool for randomized trials (RoB2) [22]. Depending on the intervention effect of interest, the studies were divided into intention-to-treat effect [29, 30, 32–34] or per-protocol effect [27, 28, 31]. Overall bias was reported as low risk in 20% of studies with an intention-to-treat analysis and 33.3% in those with a per-protocol approach. Supplementary Figure S1.

### Effect of physical activity on global cognition, subgroup analysis and sensitive analysis

The ES of multicomponent physical exercise on global cognition in MCI and dementia was 0.34 (95% IC: 0.08, 0.60). We found a moderate inconsistence (I^2^ = 41.21%). Between-trials heterogeneity was considered moderate (τ^2^ = 0.06).

**Fig. 2:**
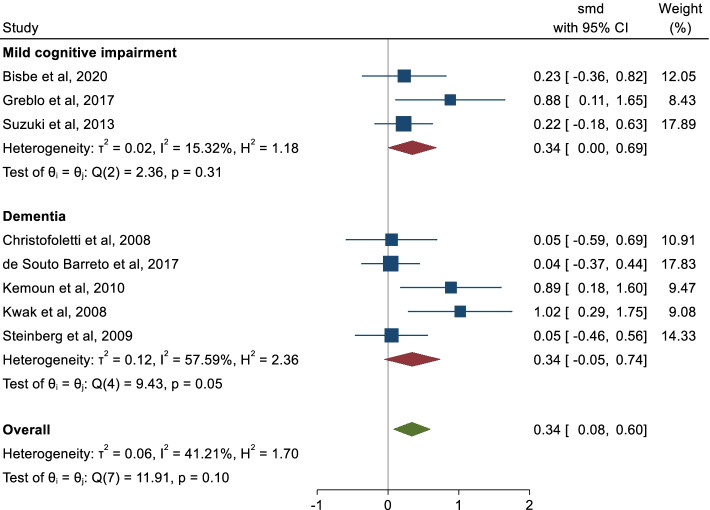
Subgroup analysis by the type of cognitive impairment

**Fig. 3:**
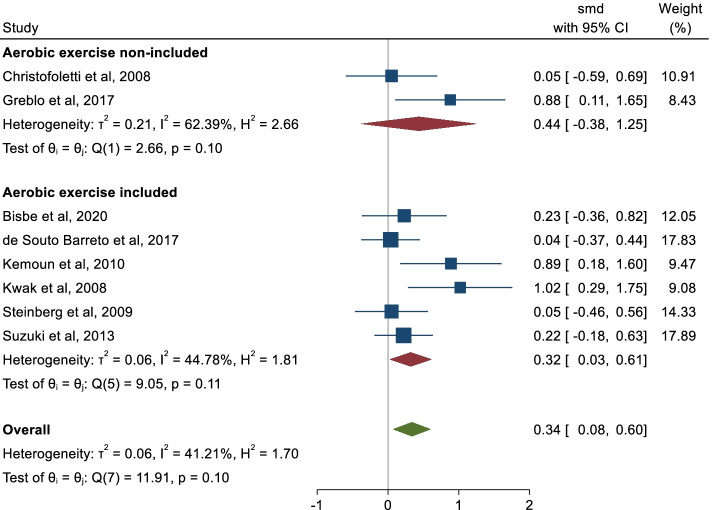
Subgroup analysis of the inclusion or not of aerobic exercise

For the subgroup analysis considering the type of cognitive impairment, the pooled ES was 0.34 (95% CI: -0.05, 0.74; I2 = 57.59%) for dementia and 0.34 (95% CI: 0.00, 0.69; I2 = 15.52%) for MCI. For the subgroup analysis by the inclusion of aerobic exercises in the exercises protocol, the ES was 0.32 (95% CI: 0.03, 0.61; I2 = 44.78%) for those multicomponent physical exercise including aerobic exercise and 0.44 (95% CI: -0.38, 1.25; I2 = 62.39%) for those not including aerobic exercise, respectively (Figs. 2 and 3).

**Fig. 4:**
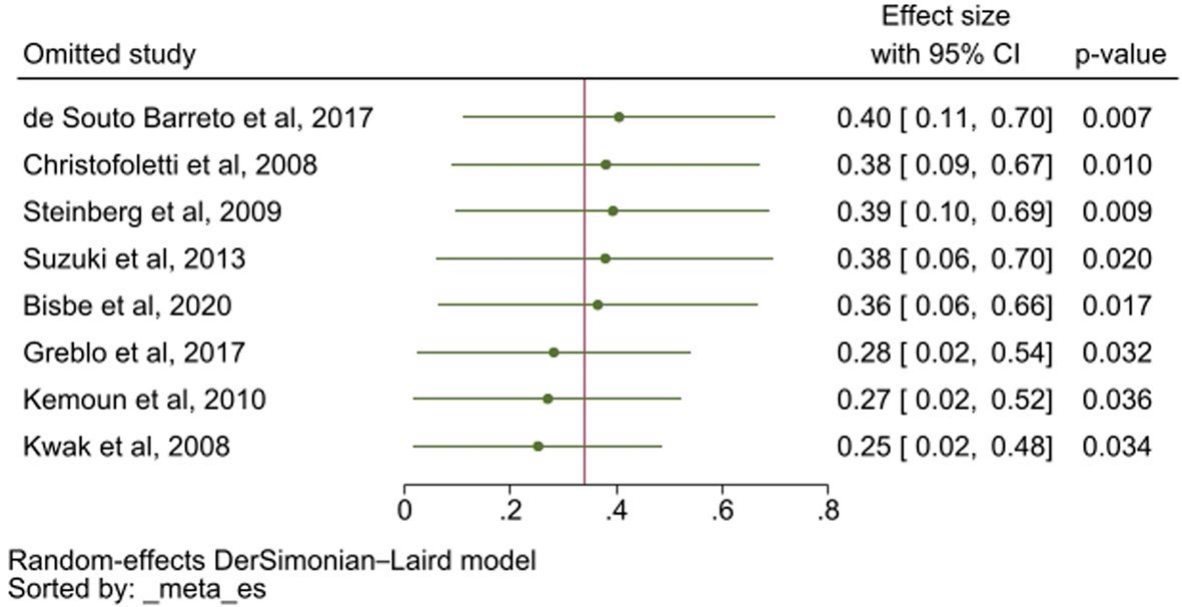
Sensitivity analysis

When sensitivity analysis was performed the two studies which omission showed a higher impact on the overall effect size were de Souto Barreto et al. (ES: 0.4; 95% CI: 0.11–0.7) and Kwak et al. (ES: 0.25; 95% CI: 0.02–0.48). Omitting the study of Kemoun et al., the only study that did not include strength exercises in the intervention, the effect size was 0.27 (95% CI: 0.02–0.52). (Fig. 4).

### Small study effects

After visual inspection of the funnel plot and considering the Eger test, we found small study effects for the effect of multicomponent physical exercise on global cognition in MCI and dementia (*p* = 0.009). The trim-and-fill analysis showed that only one study was needed to remove the small study effects and that the ES of observed and imputed studies would be 0.274 (95% CI: -0.01, 0.56) (Supplementary Figure S2).

## Discussion

This systematic review and meta-analysis provides evidence supporting the effectiveness of multicomponent physical exercise associated with aerobic exercise on global cognition in people with MCI or dementia. Furthermore, this meta-analysis shows that when exercises protocol did not include aerobic exercise this effect disappeared. Also, we did not find an effect when MCI and dementia were separately analyzed.

Our data suggest that multicomponent physical activity plus aerobic exercise produces positive effects on global cognition in patients with MCI and dementia. However, only two studies did not include aerobic exercise as part of the intervention and presented a high heterogeneity between them. Currently, multicomponent physical exercise is the one most frequently recommended for high-prevalent conditions in older adults such as frailty [35]. Although the relationship between this type of exercise and cognitive functions is unclear, the effectiveness of physical exercise in reducing frailty and improving cognition, emotions, and social networks has been reported among frail older adults [16], using multicomponent exercise and including aerobic exercise. This fact makes difficult to isolate the effect of the multicomponent exercise intervention approach, whose evidence on cognition remains inconclusive [36], as well as the most suitable combination of components, duration and dose [37]. Nevertheless and considering that physical activity is part of the intervention of the vast majority of conditions that affect older people, the inclusion of an individualized physical exercise in the management of the older patients should be part of the daily clinical practice [38].

The inclusion of aerobic exercise within the multicomponent physical exercise programs seems to be of importance in positively impacting cognitive function. The positive effect of aerobic exercise has been reported among cognitively healthy individuals [39], and appears to be stronger as age increases, suggesting a protective effect against age-related cognitive decline. Additionally, aerobic exercise has demonstrated to positively impact on the cognitive function, behavior, and mobility [40] of patients with dementia and the global cognitive ability of older adults with MCI [10], although some individual domains are not significantly improved (i.e. attention, verbal fluency, and visuospatial domains).

Some mechanisms have been proposed to explain the effects of multicomponent physical exercise on cognitive function. The association between multicomponent physical exercise (a composite strength and balance training program associated with a walking recommendation) and brain-derived neurotrophic factor (BDNF), a factor that promotes the growth and differentiation of neurons and supports the survival of existing neurons, was previously explored. It seems that blood levels of BDNF are not affected by the multicomponent physical exercise intervention [41]. This result is consistent with animal models in which strength exercise reduces aerobic exercise-induced adult hippocampal neurogenesis due to reduced BDNF and β-hydroxybutyrate [42], and with a study in young male participants that showed no effect of acute strength exercise on plasma levels of BDNF [43]. Similarly, a meta-analysis that assessed the effect of physical activity on BDNF did not find the effect of strength exercises on BDNF levels [21]. These results suggest that the multicomponent physical exercise, or at least the strength exercises, could have no effect on the neurogenesis processes mediated by BDNF. However, the BDNF is not the only cytokine with a role in the neurogenesis and neuroplasticity process. Ciliary neurotrophic factor (CNTF), leukaemia inhibitory factor (LIF), vascular endothelial growth factor (VEGF) family, and insulin-like growth factor I (IGF-I) are some cytokines that have been related to neuronal plasticity [44]. IGF-I level is related to neurotransmission, neuronal plasticity and neurotrophic potential, and the declined serum levels have been associated with age-related cognitive decline [45]. In addition, healthy levels of IGF-I are associated with an increase in hippocampal mass and verbal recall[44]. Related to physical activity, a contraction-induced muscle release of IGF-I has been shown, independently of the levels of Growth Hormone [46]. Moreover, muscle contraction and physical exercise have a relation to an improvement in mitochondrial function, that is related to a neuroprotective role through both brain plasticity and angio-neurogenesis ways [18]. Finally, resistance training showed a positive effect on spatial memory in animal models, although it has not an effect on the reduction in oxidative parameter levels [47]. This evidence suggests that the impact of multicomponent physical exercise in cognition implies different paths and supports the existence of a complex muscle-brain axis.

This study shows the importance of included a physical exercise intervention in the management of patients with cognitive decline including both aerobic and multicomponent exercise (i.e., endurance, strength, balance, or flexibility exercises), independently of the stage of cognitive impairment. As far as we know, this is the first systematic review and meta-analysis that explores the effect of multicomponent physical exercise intervention on cognition in patients with MCI and dementia, using the accepted definition of multicomponent physical exercise in the inclusion criteria. However, our study has some limitations. First, no information on independent cognitive domains was found, so only data on global cognition was reported. Second, few primary studies were found with a small sample size and therefore small study effects was found in the analysis. Third, only two studies did not include aerobic exercise as part of the intervention and presented a high heterogeneity between them. Finally, only published papers in English and Spanish were included, so no information on other languages was included.

## Conclusion

This systematic review and meta-analysis suggests a positive effect of the multicomponent physical exercises on global cognition in people with MCI or dementia specially when aerobic exercise was included in the exercise protocol. However, due to the limitations of the included studies, these findings should be cautiously interpreted. Well-designed clinical trials comparing aerobic exercise to multicomponent exercise should be conducted to clarify the true effect on cognition. However, our results support the needed to include physical exercise in the cognitive rehabilitation protocols and cognitive impairment therapies.

## Supplementary Information

**Additional file 1: Supplementary Table S1.** PubMed search strategy until January 2021. Analogous search strategies were used for Scopus, and Cochrane Database. **Supplementary Table S2**. Articles excluded. **Supplementary Figure S1.** Risk of bias using the Cochrane Collaboration´s tool for assessment of risk of bias (RoB2): A. Intention-to-treat effect; B. Per-protocol effect. **Supplementary Figure S2.** Publication bias.

## Data Availability

The datasets generated and/or analysed during the current study are available in file *data_set_srma.dta* of the repository *bmc_g-data-set* (https://github.com/lcvenegas/bmc_g-data-set).
