# Supplementary material for: Effect of multicomponent exercise in cognitive impairment: a systematic review and meta-analysis
Source: BMC Geriatr. 2022 Jul 25;22:617. doi: 10.1186/s12877-022-03302-1 (PMC9316334; doi:10.1186/s12877-022-03302-1)

| (dement* OR “cognitive dysfunction” OR “cognitive decline” OR “mild cognitive impairment” OR “cognitive impairment” OR Alzheimer* OR “Alzheimer’s disease” OR “Alzheimer’s dementia” OR “Alzheimer disease” OR “Alzheimer dementia” OR “vascular cognitive impairment” OR “vascular dementia” OR “vascular cognitive dysfunction” OR “lewy body dementia” OR “lewy body disease” OR “dementia lewy bodies” OR “dementia lewy” OR “frontotemporal dementia” OR “frontotemporal lobar degeneration” OR “pick disease”)  *AND*  (“aerobic exercise training” OR “aerobic intervention” OR “aerobic training” OR “aerobic exercise” OR strength OR “strength exercise” OR “strength training” OR “muscle strength training” OR “resistance training” OR “resistance exercise” OR “balance exercise” OR balance OR “balance training” OR “physical exercise” or “physical activity” OR endurance OR “endurance training” OR “endurance performance” OR “muscular endurance” OR “multimodal exercise” OR “multimodal intervention” OR “multimodal physical therapy” OR “multicomponent exercise” OR “multimodal physical activity” OR “multicomponent training” OR “multicomponent intervention” OR “multicomponent physical activity”)  *AND*  (“clinical trial”) |
| --- |
|  |
|  |

**Supplementary Table S1.** PubMed search strategy until January 2021. Analogous search strategies

were used for Scopus, and Cochrane Database.

**Supplementary Table S2**: Articles excluded

| **Reason to exclusion** | **Ref.** |
| --- | --- |
| Control group non reported | ^1,2^ |
| Mixed intervention (physical exercise in addition to other) | ^3–11^ |
| Cognitive assessment not performed | ^12–16^ |
| Only one type of physical exercise was included | ^17–23^ |

**Reference:**

1. Arrieta H, Rezola-Pardo C, Echeverria I, et al. Physical activity and fitness are associated with verbal memory, quality of life and depression among nursing home residents: Preliminary data of a randomized controlled trial. *BMC Geriatr*. 2018;18(1). doi:10.1186/s12877-018-0770-y

2. Kwak Y-S, Um S-Y, Son T-G, Kim D-J. Effect of regular exercise on senile dementia patients. *Int J Sports Med*. 2008;29(6):471-474. doi:10.1055/s-2007-964853

3. Baker R, Bell S, Baker E, et al. A randomized controlled trial of the effects of multi-sensory stimulation (MSS) for people with dementia. *Br J Clin Psychol*. 2001;40(1):81-96. doi:10.1348/014466501163508

4. Dannhauser TM Whitfield TJ, Fletcher BC, Stevens T, Walker Z CM. A complex multimodal activity intervention to reduce the risk of dementia in mild cognitive impairment-ThinkingFit: pilot and feasibility study for a randomized controlled trial. *BMC Psychiatry*. 2014;14(1). http://ovidsp.ovid.com/ovidweb.cgi?T=JS&PAGE=reference&D=cctr&NEWS=N&AN=CN-00992782

5. De Andrade LP, Gobbi LTB, Coelho FGM, Christofoletti G, Riani Costa JL, Stella F. Benefits of multimodal exercise intervention for postural control and frontal cognitive functions in individuals with Alzheimer’s disease: A controlled trial. *J Am Geriatr Soc*. 2013;61(11):1919-1926. doi:10.1111/jgs.12531

6. de Souto Barreto P Rolland Y, Vellas B AS. Physical activity domains and cognitive function over three years in older adults with subjective memory complaints: secondary analysis from the MAPT trial. *J Sci Med Sport*. 2017;(no pagina. http://ovidsp.ovid.com/ovidweb.cgi?T=JS&PAGE=reference&D=cctr&NEWS=N&AN=CN-01403254

7. Gates NJ, Valenzuela M, Sachdev PS, et al. Study of Mental Activity and Regular Training (SMART) in at risk individuals: A randomised double blind, sham controlled, longitudinal trial. *BMC Geriatr*. 2011;11. doi:10.1186/1471-2318-11-19

8. Hagovská M, Olekszyová Z. Impact of the combination of cognitive and balance training on gait, fear and risk of falling and quality of life in seniors with mild cognitive impairment. *Geriatr Gerontol Int*. 2016;16(9):1043-1050. doi:10.1111/ggi.12593

9. Lam LCW, Chau RCM, Wong BML, et al. A 1-Year Randomized Controlled Trial Comparing Mind Body Exercise (Tai Chi) With Stretching and Toning Exercise on Cognitive Function in Older Chinese Adults at Risk of Cognitive Decline. *J Am Med Dir Assoc*. 2012;13(6):568.e15-568.e20. doi:10.1016/j.jamda.2012.03.008

10. Lam LC-W, Chan WC, Leung T, Fung AW-T, Leung EM-F. Would older adults with mild cognitive impairment adhere to and benefit from a structured lifestyle activity intervention to enhance cognition?: a cluster randomized controlled trial. *PLoS One*. 2015;10(3):e0118173. doi:10.1371/journal.pone.0118173

11. Pereira C, Rosado H, Cruz-Ferreira A, Marmeleira J. Effects of a 10-week multimodal exercise program on physical and cognitive function of nursing home residents: A psychomotor intervention pilot study. *Aging Clin Exp Res*. 2018;30(5):471-479. doi:10.1007/s40520-017-0803-y

12. Bürge E, Berchtold A, Maupetit C, et al. Does physical exercise improve ADL capacities in people over 65 years with moderate or severe dementia hospitalized in an acute psychiatric setting? A multisite randomized clinical trial. *Int Psychogeriatrics*. 2017;29(2):323-332. doi:10.1017/S1041610216001460

13. Hauer K, Ullrich P, Dutzi I, et al. Effects of Standardized Home Training in Patients with Cognitive Impairment following Geriatric Rehabilitation: A Randomized Controlled Pilot Study. *Gerontology*. 2017;63(6):495-506. doi:10.1159/000478263

14. Nascimento CMC, Teixeira CVL, Gobbi LTB, Gobbi S, Stella F. A controlled clinical trial on the effects of exercise on neuropsychiatric disorders and instrumental activities in women with Alzheimer’s disease [Efeitos do exercício físico sobre distúrbios neuropsiquiátricos e atividades instrumentais da vida diária e. *Brazilian J Phys Ther*. 2012;16(3):197-204. doi:10.1590/S1413-35552012005000017

15. Fleiner T Gersie M, Zijlstra W, Haussermann P DH. Structured physical exercise improves neuropsychiatric symptoms in acute dementia care: a hospital-based RCT. *Alzheimers Res Ther*. 2017;9(1). http://ovidsp.ovid.com/ovidweb.cgi?T=JS&PAGE=reference&D=cctr&NEWS=N&AN=CN-01415786

16. Liu Z Trombetti A, King AC, Liu CK, Manini TM, Fielding RA, Pahor M, Newman AB, Kritchevsky S, Gill TM, LIFE Study investigators HFC. Effect of 24-month physical activity on cognitive frailty and the role of inflammation: the LIFE randomized clinical trial. *BMC Med*. 2018;16(1):185. http://ovidsp.ovid.com/ovidweb.cgi?T=JS&PAGE=reference&D=cctr&NEWS=N&AN=CN-01650402

17. Holthoff VA, Marschner K, Scharf M, et al. Effects of Physical Activity Training in Patients with Alzheimer’s Dementia: Results of a Pilot RCT Study. Quinn TJ, ed. *PLoS One*. 2015;10(4):e0121478. doi:10.1371/journal.pone.0121478

18. Lamb SE, Sheehan B, Atherton N, et al. Dementia And Physical Activity (DAPA) trial of moderate to high intensity exercise training for people with dementia: Randomised controlled trial. *BMJ*. 2018;361. doi:10.1136/bmj.k1675

19. Langoni Chandra da S, Resende Thais de L, Barcellos Andressa B, et al. Effect of exercise on cognition, conditioning, muscle endurance, and balance in older adults with mild cognitive impairment: a randomized controlled trial. *J Geriatr Phys Ther 2019 Apr-Jun;42(2)E15-E22*. 2019;42(2):E15-E22. doi:10.1519/JPT.0000000000000191

20. Lautenschlager NT, Cox KL, Flicker L, et al. Effect of Physical Activity on Cognitive Function in Older Adults at Risk for Alzheimer Disease. *JAMA*. 2008;300(9):1027. doi:10.1001/jama.300.9.1027

21. Scherder EJA, Van Paasschen J, Deijen J-B, et al. Physical activity and executive functions in the elderly with mild cognitive impairment. *Aging Ment Health*. 2005;9(3):272-280. doi:10.1080/13607860500089930

22. Vreugdenhil A, Cannell J, Davies A, Razay G. A community-based exercise programme to improve functional ability in people with Alzheimer’s disease: A randomized controlled trial. *Scand J Caring Sci*. 2012;26(1):12-19. doi:10.1111/j.1471-6712.2011.00895.x

23. Yoon DH, Lee J-Y, Song W. Effects of Resistance Exercise Training on Cognitive Function and Physical Performance in Cognitive Frailty: A Randomized Controlled Trial. *J Nutr Heal Aging*. 2018;22(8):944-951. doi:10.1007/s12603-018-1090-9

**Supplementary Figure S1.**  Risk of bias using the Cochrane Collaboration´s tool for assessment of risk of bias (RoB2): A. Intention-to-treat effect; B. Per-protocol effect.

A.

B.

**Supplementary Figure S2.** Publication bias


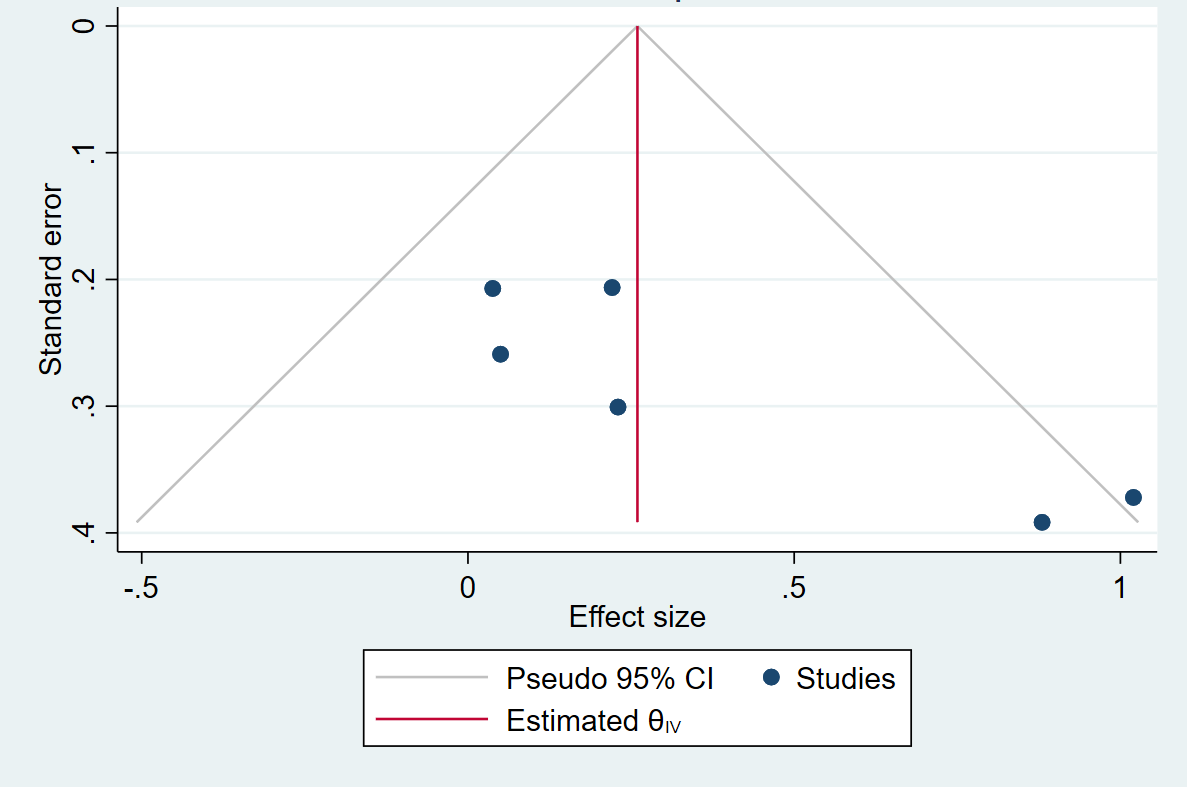

Supplement: Supplementary file 1 — Additional file 1: Supplementary Table S1. PubMed search strategy until January 2021. Analogous search strategies were used for Scopus, and Cochrane Database. Supplementary Table S2. Articles excluded. Supplementary Figure S1. Risk of bias using the Cochrane Collaboration´s tool for assessment of risk of bias (RoB2): A. Intention-to-treat effect; B. Per-protocol effect. Supplementary Figure S2. Publication bias. [file 12877_2022_3302_MOESM1_ESM.docx]
